# Glial Purinergic Signaling in Neurodegeneration

**DOI:** 10.3389/fneur.2021.654850

**Published:** 2021-05-14

**Authors:** Marie J. Pietrowski, Amr Ahmed Gabr, Stanislav Kozlov, David Blum, Annett Halle, Kevin Carvalho

**Affiliations:** ^1^Microglia and Neuroinflammation Laboratory, German Center for Neurodegenerative Diseases (DZNE), Bonn, Germany; ^2^Department of Physiology, Faculty of Veterinary Medicine, Cairo University, Giza, Egypt; ^3^University of Lille, Inserm, CHU Lille, U1172 LilNCog - Lille Neuroscience and Cognition, Lille, France; ^4^Alzheimer and Tauopathies, Labex DISTALZ, Lille, France; ^5^Institute of Neuropathology, University of Bonn, Bonn, Germany

**Keywords:** Alzheimer, neurodegeneration, ATP, adenosine, oligodendrocyte, microglia, astrocyte, purine

## Abstract

Purinergic signaling regulates neuronal and glial cell functions in the healthy CNS. In neurodegenerative diseases, purinergic signaling becomes dysregulated and can affect disease-associated phenotypes of glial cells. In this review, we discuss how cell-specific expression patterns of purinergic signaling components change in neurodegeneration and how dysregulated glial purinergic signaling and crosstalk may contribute to disease pathophysiology, thus bearing promising potential for the development of new therapeutical options for neurodegenerative diseases.

## Introduction

Neurodegenerative diseases are characterized by a progressive loss of structure and function of the CNS. An estimated 50 million patients worldwide are currently affected by neurodegenerative diseases and it has been projected that this number will rise to 131 million patients by 2050 (1). Many of these diseases are idiopathic/polygenic proteinopathies, which are characterized by accumulation and/or aggregation of proteins such as TAU, amyloid-β or α-synuclein (α-SYN) in the CNS and are multifactorial, with both genetic and environmental risk factors. The most common ones are the sporadic forms of Alzheimer's disease (AD; TAU/amyloid-β (Aβ) aggregates), Parkinson's disease (PD; α-SYN aggregates), amyotrophic lateral sclerosis (ALS; TAR DNA binding protein 43 (TDP-43)/FUS RNA binding protein (FUS) aggregates), Lewy body dementia (α-SYN aggregates) and frontotemporal lobar degeneration (FTLD; TAU/TDP-43/FUS aggregates) (2).

Despite decades of research, no causal treatment is available for any of these diseases. One promising approach to help overcome the lack of therapeutical options is to shift the focus from a linear neuron-centered view on how neurodegenerative diseases develop to a broader and integrative view in which interactions between all cell types in the brain are considered. This paradigm shift has led to an increased interest in the role of glial cells in neurodegeneration.

Indeed, there is strong evidence that changes in glial cells, microglia, astrocytes and oligodendrocytes, are causally involved in neurodegenerative diseases. Around half of the known genetic risk factors in sporadic AD are glial genes related to immune function such as *TREM2, CD33* or *CR1* (3, 4). Furthermore, recent data have highlighted phenotypic changes of glial cells over the disease course with the discovery of disease-associated microglial (DAM) and disease-associated astrocytic markers (DAA) (5, 6). Accordingly, new therapeutic strategies targeting glial signaling pathways are currently being tested in pre-clinical intervention studies, for example, treatments modulating the NLRP3 inflammasome (7), astrocytic nuclear factor kappa-β (8) and TREM2-mediated signaling (9), among others.

The targeting of purinergic signaling—i.e., signaling pathways mediated by extracellular nucleotides and nucleosides such as ATP or adenosine—belongs to these novel emerging therapeutic strategies (10). All CNS cell types, including glial cells, express purinergic receptors and purinergic signaling influences key CNS functions [for review see Agostinho et al. (11)] such as synaptic transmission, proliferation, maturation and neuroinflammation (12) that are altered in neurodegeneration. Thus, in the present manuscript, we discuss the current knowledge on purinergic signaling in glial cells and its potential relevance in neurodegenerative disease. We provide a comprehensive overview and cell-specific expression tables based on available transcriptomic data of purinergic genes in glial cells in neurodegeneration and link this data with data from functional studies. Finally, we discuss glial purinergic signaling as a potential target for future therapeutic intervention.

## The Components of Purinergic Signaling

Purines are a family of small molecules involved in DNA/RNA structure and key cellular processes such as cell metabolism, intracellular signaling and extracellular signaling. Geoffrey Burnstock coined the term of “purinergic signaling” for the latter in 1972 (13), in which he referred to cell signaling pathways that are activated by engagement of nucleosides and nucleotides with specific cell receptors. ATP, the main energy storage of the cell, can be hydrolyzed in the extracellular space through specific enzymes called ectonucleotidases into ADP, AMP and adenosine. These include ectonucleoside triphosphate diphosphohydrolases (E-NTPDases), ectonucleotide pyrophosphatase/ phosphodiesterases (ENPPs), alkaline phosphatases and ecto-5′-nucleotidase (*NT5E*/CD73) (14). Adenosine can also be produced through the S-adenosyl-L-homocysteine pathways and degraded by adenosine deaminase and adenosine kinase. Furthermore, ATP and its derivatives can be transported between cell compartments and released extracellularly through different transporters like the equilibrative nucleoside transporters (ENTs) or concentrative nucleoside transporters (CNTs), but also through channels such as Connexin-43 (CX43), Connexin-32 (CX32) or Pannexin-1 (PANX1), secretion involving vesicular nucleotide transporter (*SLC17A9/*VNUT) or ultimately by membrane rupture during cellular injury, allowing them to trigger purinergic receptor signaling (15, 16).

Purinergic receptors are subclassified into two large families: P1 receptors that are activated by nucleosides, and P2 receptors that are activated by nucleotides. P2 receptors are further subdivided into ionotropic P2X receptors and metabotropic P2Y receptors (17, 18). So-called P0 receptors have recently been defined and constitute adenine receptors (19, 20). The P1 receptor family consists of four receptors (adenosine A1, A2A, A2B and A3 receptor), the P2X family of seven receptors (P2X1-P2X7) and the P2Y family of eight receptors (P2Y1, P2Y2, P2Y4, P2Y6, P2Y11-P2Y14).

## Glial Purinergic Signaling and Crosstalk Under Physiological Conditions

**Figure 1:**
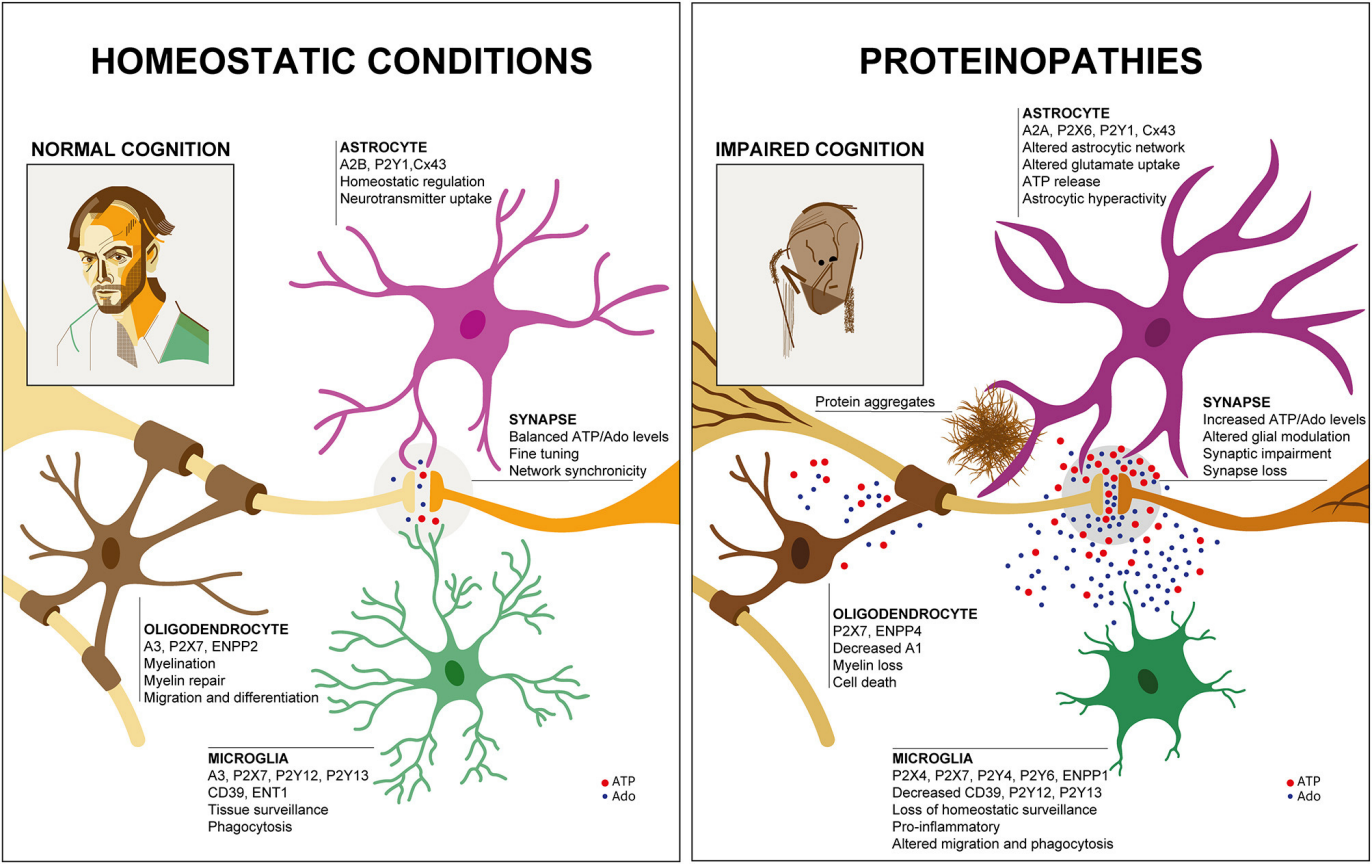
Glial purinergic signaling under homeostatic conditions and in proteinopathies. Under healthy conditions **(Left)**, cell-specific glial purinergic signaling contributes to fine-tuning of synapse function and thus, normal cognitive abilities. In proteinopathies **(Right)**, accumulation of protein aggregates leads to glial phenotype changes that are associated with altered expression of purinergic signaling components and altered ATP and adenosine (Ado) levels. This impacts synaptic function and ultimately results in synapse loss, contributing to impaired cognition. Two self-portraits are shown to illustrate the cognitive status in health and disease, similar to the famous work of William Utermohlen, who continued to create self-portraits after being diagnosed with AD.

**Figure 2:**
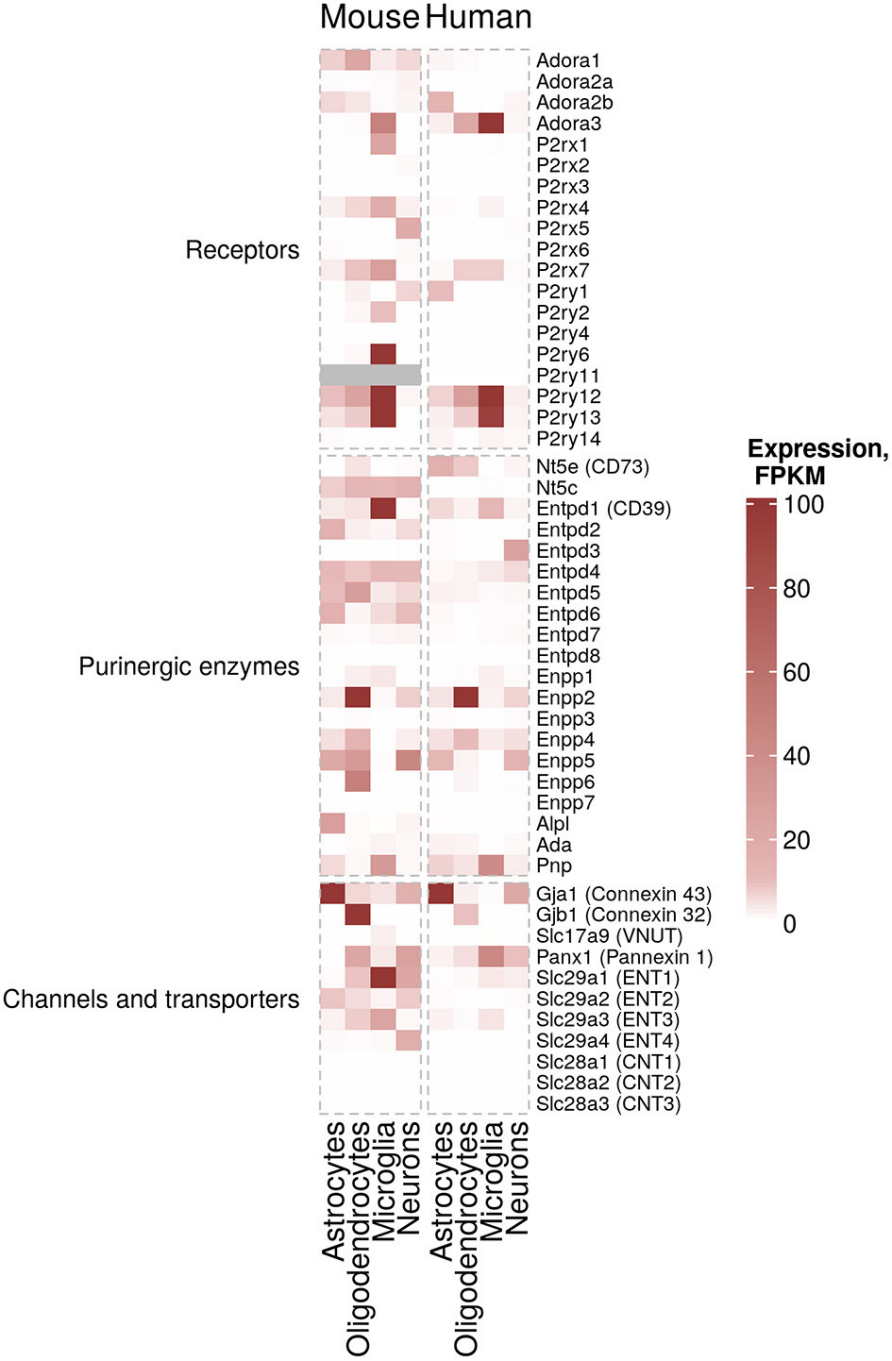
Cell-specific expression of purinergic genes. Estimated expression levels FPKM (Fragments Per Kilobase Million) of genes involved in purinergic signaling pathways in microglia, myelinating oligodendrocytes, mature astrocytes and neurons was obtained from existing data sets from Zhang et al. (30) (mouse data) and Zhang et al. (31) (human data) (30, 31). FPKM values of 100 and above are shown in the same color for visualization purposes.

Purinergic signaling controls important physiological processes in the healthy CNS, such as synaptic transmission, cell proliferation and innate immune response (11, 12). ATP and adenosine are produced and released upon neuronal or glial activation and initiate various cellular pathways corresponding to the activation of P2X, P2Y and adenosine receptors. Neurons, microglia, astrocytes and oligodendrocytes express a unique repertoire of purinergic receptors (Figure 1, left), which due to receptor-specific intracellular downstream signaling cascades lead to specific responses in each CNS cell type. Many previous studies have contributed to our understanding of the purinergic signaling repertoires in microglia (21–24), astrocytes (25–27) and oligodendrocytes (28, 29). However, a full overview of the expression patterns of all components of purinergic signaling in the main mouse and human CNS cells has only become available through the seminal work of the laboratory of Ben Barres. The group created an unbiased RNA sequencing database of purified specific CNS cells from human and mouse cortex (30–32). Using this database, we here assembled expression data of purinergic genes from neurons, microglia, astrocytes and oligodendrocytes. With the caveat of missing validation on the protein level, possible cell impurities, region-specific differences or age-dependent modifications, this makes it possible to compare expression patterns of purinergic genes in these CNS cell types in human and mouse (Figure 2).

According to this data, microglia are the cells with the highest expression and largest number of expressed purinergic receptors (in particular *ADORA3, P2RY12, P2RY13*, and at lower levels *P2RX7* and *P2RY14* genes) and ectonucleotidases (in particular *ENTPD1* gene also known as CD39) in the human cortex. There are significant differences in murine microglial gene expression of purinergic components compared to human. For example, *Adora1, P2rx4* and *Entpd2* show higher expression levels in mouse. Additionally, genes encoding ENTs (ENT1, 2 and 3, encoded by *Slc29a1, Slc29a2* and *Slc29a3*, correspondingly) are expressed at higher levels in mouse microglia, suggesting that nucleoside transport processes may differ between humans and mice. Similarly, cell-specific expression of some ectonucleotidases, including *Entpd1* in microglia are considerably different in human and mouse (Figure 2).

Human astrocytes, despite displaying–in some cases considerably–lower expression values than microglia, also express a variety of purinergic receptors (*ADORA2B, P2RY1, P2RY12*) and ectonucleotidases (*ENTPD1, ENPP5* and *NT5E* also known as CD73). Similar patterns can be found in mouse astrocytes (Figure 2).

Lastly, human oligodendrocytes express *ADORA3, P2RX7, P2RY12, P2RY13*, and *NT5E*. Interestingly, ectonucleotide pyrophosphatases/phosphodiesterases (ENPPs, namely *Enpp2, Enpp4, Enpp5*, and *Enpp6*) in oligodendrocytes show a higher expression in mouse (Figure 2).

Although some of the data largely overlaps with previous findings in rodent and human CNS tissue, this cell type-specific gene expression table allows to better appreciate the considerable inter-species similarities–and differences–between human and mouse. It also shows the large spectrum of purinergic gene expression patterns in the different CNS cell types.

How is this cell type-specific purinergic signaling repertoire linked to glial cell function? Although some aspects have remained ill-defined, especially regarding the role of purinergic enzymes and transporters, a lot is known about how purinergic receptor signaling shapes glial cell function in the healthy brain [Figure 1 left; for a recent detailed review see Agostinho et al. (11)].

For example, different P1 and P2 receptors such as A3, P2Y12, P2Y13, P2X4 control the motility of the ramified and dynamic cell processes of microglia, the tissue-resident immune cells of the brain (33–36). This motility of cell processes is important for surveillance and chemotactic cell process movement toward localized brain damage. Furthermore, phagocytosis and release of inflammatory molecules, both major effector functions of microglia, are modulated by purinergic signaling pathways, involving P2X4, P2X7, and P2Y6 (37–40). In addition, there is evidence that microglial contribution to synaptic pruning and synaptic function is at least partly controlled by purinergic mechanisms (41). It has also been shown that innate immune responses of microglia are positively modulated by ATP through P2X7 receptors (42) and negatively by adenosine through A2A receptors (43).

Astrocytes are involved in a wide variety of functions in the CNS, such as metabolic support, synaptic function, neuronal and synaptic maturation and blood brain barrier permeability (44–47). The relevance of astrocytic P2 receptor signaling in some of these cell functions has been implicated in a number of previous studies. More specifically, P2Y1 mediates calcium signaling in astrocytes, which is critical for modulating synapse function and blood-brain barrier maintenance (48). Neurotransmitter recapture is also modulated by astrocytic A2A receptor-mediated signaling, allowing for a reduced uptake of glutamate and greater uptake of GABA, reinforcing synaptic activation (49, 50).

Oligodendrocytes synthesize myelin sheaths, which insulate CNS axons, enabling a rapid action potential propagation. This process occurs life-long as oligodendrocytes perform myelin remodeling. Additionally, oligodendrocytes contribute to the metabolic support of axons through a wide variety of transporters (51). Oligodendrocyte progenitor cells (OPC, also called NG2 cells as they express the NG2 chondroitin sulfate proteoglycan) can differentiate into new mature oligodendrocytes. As for microglia and astrocytes, there is evidence that purinergic signaling controls a number of cell functions in oligodendrocytes. Adenosine and ATP/ADP have been shown to contribute to proliferation, migration and maturation of oligodendrocytes through P1 (A1 and A2A) and P2 receptors (P2X7 and P2Y1).

Apart from its importance in shaping cell functions of particular glial cells, purinergic signaling plays a key role in neuro-glial interactions (11). Neurons and glial cells reside in spatial proximity to each other, which is particularly important at the synaptic site. This proximity enables an efficient inter-cellular crosstalk through purinergic signaling, based on ATP/adenosine release, ectonucleotidase activity and receptor stimulation, affecting the neighboring cells in an autocrine and paracrine manner.

For example, ATP and adenosine released by synaptic stimulation trigger calcium wave signaling in the astrocytic syncytium through P2Y receptors, which in response decrease the activation of neighboring synapses through adenosine A1 receptor, a phenomenon called “heterosynaptic depression” (52, 53). Moreover, neuronal ATP release at the synapse recruits microglial cell processes, allowing cleavage of ATP into adenosine through microglial CD39 and ubiquitous CD73, leading to dampening of synapse activity upon A1 receptor stimulation (41).

Furthermore, ectonucleotidases such as CD73 are required for adenosine A2A receptor potentiation of synapses, and such mechanism could be similar in a glial context (54). For instance, *ENTPD1* (CD39) and *NT5E* (CD73) are expressed in all CNS cell types in the human CNS, albeit in case of CD73 at very low and in case of CD39 at high levels in microglia compared to the other cell types (31). This suggests that local degradation of ATP and ADP and production of adenosine may also occur in a cell-autonomous manner.

## Glial Purinergic Signaling and Crosstalk in Neurodegeneration

Since purinergic signaling is a multi-cellular, dynamic and complex signaling system, some of its aspects in neurodegenerative diseases have been difficult to evaluate and will require more specific quantitative tools in the future.

For instance, one major question that has remained unresolved due to the lack of specific tools with sufficient temporal and spatial resolution, is how the spatio-temporal kinetics of ligand availability and purinergic receptor activation is regulated in neurodegeneration (55). However, it has recently been shown that purinergic metabolites are strongly modified in AD. Adenosine was shown to be the most affected purine, increasing in temporal and parietal cortices of AD brains (56). Similarly, increased levels of adenosine are detectable in the CSF of ALS patients (57).

**Figure 3:**
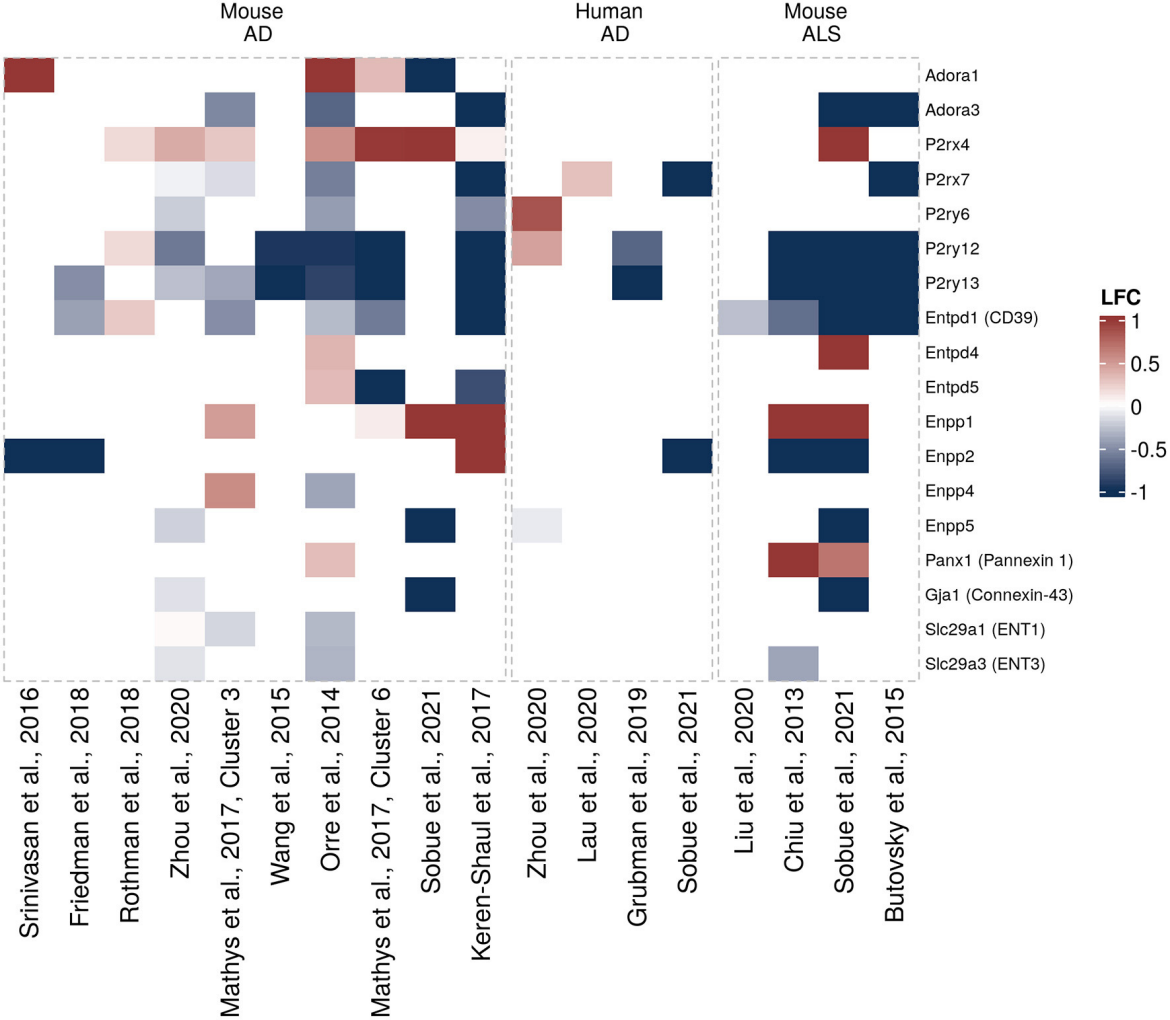
Differentially expressed purinergic genes in microglia in neurodegeneration. Heatmap shows Log2 fold changes in expression of genes involved in purinergic signaling pathways between disease (AD or ALS, or relevant mouse models) and control samples. LFC values above 1 and below −1 are shown in the same color intensity for visualization purposes. Studies were selected according to the following criteria: transcriptomic analysis was performed on single cells or nuclei and allows disease/control comparisons for selected cell types, or on bulk-sorted glial populations; differential expression analysis results were provided by authors as supplementary material and were accessible. In total, 18 data sets from 14 studies were included for microglia [Orre et al. (69) (mouse, AD model, bulk), Chiu et al. (63) (mouse, ALS model, bulk), Wang et al. (64) (mouse, AD model, bulk), Srinivasan et al. (65) (mouse, AD model, bulk), Friedman et al. (66) (mouse, AD model, bulk), Rothman et al. (58) (mouse, AD model, bulk), Mathys et al. (72) (mouse, AD model, single-cell, two disease-associated clusters), Keren-Shaul et al., (5) (mouse, AD model, single-cell, DAM cluster), Zhou et al. (68) (two data sets, mouse AD model and human AD patients, single-nuclei), Liu et al. (59) (mouse, ALS model, single-cell), Lau et al. (61) (human, ALS, single-nuclei), Grubman et al. (74) (human, AD, single-nuclei), Butovsky et al. (75) (mouse, ALS model, bulk), Sobue et al. (60) (three data sets, mouse AD and ALS models, and human AD patients, bulk)] (5, 58–68).

**Figure 4:**
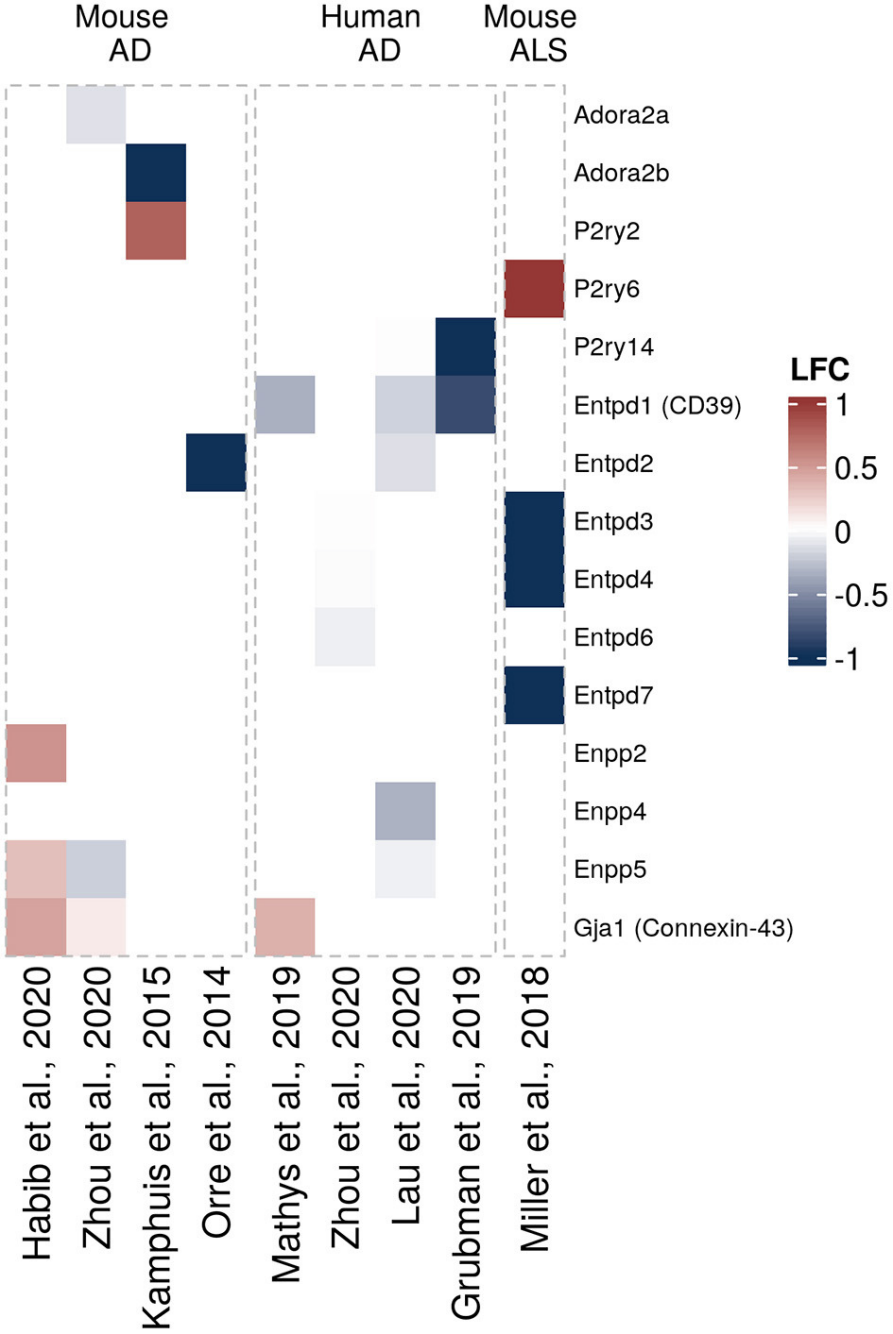
Differentially expressed purinergic genes in astrocytes in neurodegeneration. Heatmap shows Log2 fold changes in expression of genes involved in purinergic signaling pathways between disease (AD or ALS, or relevant mouse models) and control samples. LFC values above 1 and below −1 are shown in the same color intensity for visualization purposes. Studies were selected according to the following criteria: transcriptomic analysis was performed on single cells or nuclei and allows disease/control comparisons for selected cell types, or on bulk-sorted glial populations; differential expression analysis results were provided by authors as supplementary material and were accessible. In total, nine data sets from eight studies were included for astrocytes [Orre et al. (69) (mouse, AD model, bulk), Kamphuis et al. (70) (mouse, AD model, bulk), Miller et al. (71) (mouse, ALS model, bulk), Mathys et al. (72) (human, AD, single-nuclei), Zhou et al. (68) (two data sets, mouse AD model and human AD patients, single-nuclei), Habib et al. (73) (mouse, AD model, single-nuclei), Lau et al. (61) (human, ALS, single-nuclei), Grubman et al. (74) (human, AD, single-nuclei)] (61, 68–74).

**Figure 5:**
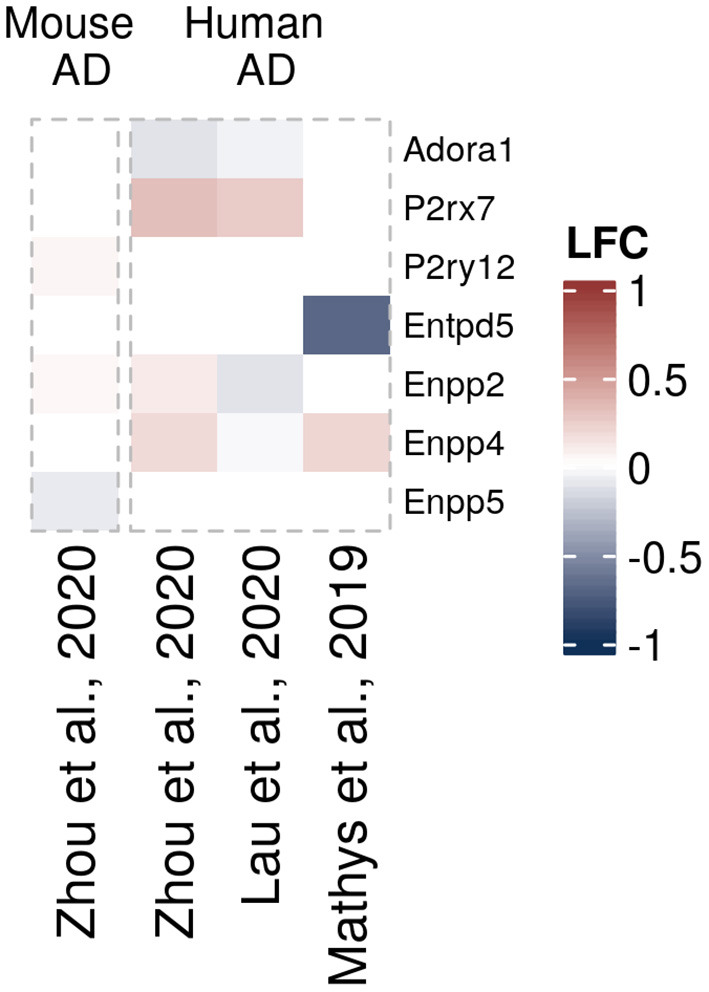
Differentially expressed purinergic genes in oligodendrocytes in neurodegeneration. Heatmap shows Log2 fold changes in expression of genes involved in purinergic signaling pathways between disease (AD or relevant mouse models) and control samples. LFC values above 1 and below −1 are shown in the same color intensity for visualization purposes. Studies were selected according to the following criteria: transcriptomic analysis was performed on single cells or nuclei and allows disease/control comparisons for selected cell types, or on bulk-sorted glial populations; differential expression analysis results were provided by authors as supplementary material and were accessible. In total, four data sets from three studies were included for oligodendrocytes [Mathys et al. (72) (human, AD, single-nuclei), Zhou et al. (68) (two datasets, mouse AD model and human AD patients, single-nuclei), Lau et al. (61) (human, ALS, single-nuclei)] (61, 68, 72).

Another question that has not been sufficiently addressed until recently has been whether and how expression of purinergic signaling components is altered in glial cells in neurodegeneration. To get a better understanding of disease-associated changes, we took advantage of the increasing number of unbiased transcriptomic studies on human and murine glial cells in neurodegeneration. These studies did not have the primary goal to investigate differential purinergic gene expression in glial cells. However, they now constitute valuable resources to detect potential patterns of purinergic transcriptomic response in microglia, astrocytes and oligodendrocytes in neurodegeneration. To assemble the available data in a comprehensive manner, we searched for studies from transgenic AD, PD, tauopathy and ALS mouse models and human AD, PD, tauopathy and ALS patients, in which transcriptomic analyses on single cells or nuclei and bulk-sorted glial populations were performed. Among the available studies that mainly covered AD and ALS, we selected the data sets, in which at least one component of purinergic signaling was significantly dysregulated. We thus included 18 data sets from 14 studies on microglia (5, 58–68), nine data sets from eight studies on astrocytes (61, 68–74) and 4 data sets from three studies on oligodendrocytes (61, 68, 72) and used log2 fold change expression values between diseased and control samples to visualize shifts of gene expression involved in purinergic signaling pathways for each cell type (Figures 3–5).

Using this data and functional studies on glial purinergic signaling, we will discuss in the following sections what is known about purinergic signaling in microglia, astrocytes and oligodendrocytes in neurodegeneration.

It should be noted, however, that glial populations in neurodegeneration are spatially and temporally heterogeneous, depending on disease stage and proximity to specific pathological hallmarks. This heterogeneity is well-described for microglial populations in AD, in which “early” and “late” subsets of disease-associated microglia occur on both gene and protein expression levels (5, 76, 77). Moreover, single-cell transcriptomic studies have shown divergent but co-existent microglial subsets with different directions of gene expression changes (67, 76, 78). Therefore, it is likely that functionally and spatially distinct glial subsets co-exist within disease-associated reactive populations of glial cells [disease-associated microglia (DAM, (5)), disease-associated astrocytes (DAA, (73)) and pathology-associated populations of oligodendrocytes (67, 79)]. This may explain some of the discrepancies between purinergic gene expression changes and evidence from functional studies.

### Microglia

Microglia are centrally involved in the pathophysiology of neurodegenerative diseases. Microglia not only drive inflammation in neurodegenerative diseases, but, as the main phagocytes in the CNS, are important for clearing protein aggregates, debris and apoptotic cells (80–86). Furthermore, the elaborate cellular morphology and process motility that enable efficient tissue surveillance are altered in neurodegeneration and this capability is therefore strongly affected (87–90). Some of these changes in microglial cellular functions may be caused by alterations in microglial purinergic signaling upon neurodegeneration (Figure 1, right).

When interrogating the microglia purinergic transcriptomic data from AD patients and AD mouse models we summarized here, a microglial response pattern emerges, in which *Adora1* and *P2rx4* are upregulated and *Adora3, P2rx7, P2ry6, P2ry12*, and *P2ry13* downregulated (Figure 3). There are also changes in ectonucleotidase expression, particularly in *Enpp1* (upregulated) and *Entpd1* (downregulated) that are consistent between several data sets in mice. However, the majority of differentially expressed genes is only detected as differentially expressed in a few studies. Some inconsistencies in expression changes of some genes including upregulation of *P2RY6* and *P2RY12* in Zhou et al. (68), or upregulation of *P2ry12* and *Entpd1* in Rothman et al. (58), may be explained by differences in the experimental approaches (58, 68). A number of ALS-related changes in gene expression are similar to gene expression patterns from AD studies, including *P2ry12* and *P2ry13* (91) that are downregulated both in murine AD and ALS mouse models. Transcriptomic data sets for glial cells in Parkinson's disease are still largely missing, both from mouse models and human brain tissue. However, there was no clear association between PD risk genes and microglia or astrocyte populations in a recent study, in which single nuclei from substantia nigra and cortex were extracted and analyzed, suggesting that microglial purinergic alterations may play a less prominent role in PD than in AD (92).

In contrast to these transcriptomic data, numerous experimental studies have shown that microglial P2X7 protein levels are elevated in human AD brains, in multiple rodent amyloidosis models (93–98), in the PD rat model 6-OHDA as well as in the spinal cord of ALS patients and SOD1 mutant mice that serve as ALS animal models (99–101).

These findings are functionally important since enhanced activity of P2X7 drives cellular inflammation in several neurodegenerative pathologies. On a molecular level, Aβ-induced ATP release activates P2X7, which in turn results in ROS production (98, 102–104) as well as activation of the inflammasome and subsequent release of the cytokine IL-1β (82, 105–108). Similarly, SOD1G93A and TDP-43Q331K in ALS and α-synuclein in PD, contribute to oxidative stress and inflammation via microglial P2X7 activation (83, 109–111). On a functional level, it was demonstrated that inhibition of P2X7 decreases migration of microglia *in vitro*, whereas phagocytosis is enhanced *in vivo* in the J20 AD model (93) making it an interesting target. The functional role of P2X7 in the pathogenesis of ALS is less clear than in AD. On the one hand, SOD1 mutant microglia show reduced ATP degradation and enhanced ATP sensitivity *in vitro* (100). On the other hand, the onset of clinical symptoms seemed to be accelerated and disease progression exacerbated (112) in P2X7-deficient SOD1 mutant mice. This indicates a complex scenario, which has implications for potential treatment strategies, as the time point of P2RX7 counteraction seems to play a key role. Microglia seem to play a dual role in ALS, acting beneficially at early stages of the disease, while exacerbating disease pathology at later stages (113). Taken together, P2RX7 activation and subsequent inflammatory response represent a common purinergic dysregulation in proteinopathies such as AD, PD and ALS. However, further research needs to be conducted, especially in the fields of ALS and PD research.

As mentioned earlier, microglial morphology, motility and chemotaxis are regulated by the interplay of multiple different purinergic receptors including P1 and P2 receptors. The adenosine A1 and A3 receptors both play a role in microglial migration (33, 114). A1 is involved in morphological changes of microglia (115) and A3 contributes to the regulation of process extension (33). Consequently, the transcriptomic changes of *Adora1* (A1) and *Adora3* (A3) that can be detected in the transcriptomic studies in neurodegeneration (Figure 3) may contribute to alterations in microglial morphology, although functional studies are currently missing. In contrast to *Adora1* and *Adora3*, expression of *Adora2a* (encoding A2A) is barely detectable under normal conditions (Figure 2). Nevertheless, A2A has been shown to mediate microglia process retraction (116), to induce *Ptgs2* (Cyclooxygenase 2) expression (117) and proliferation of microglia (118). Interestingly, A2A was found to be increased in microglia in AD when using immunolabelling (119). Consequently, this upregulation could contribute to increased microglia cell number and decreased morphology around Aβ plaques. However, microglia-specific studies investigating the effects of P1 receptors in neurodegeneration are limited and further investigations will be required.

P2Y12 and P2Y13, encoded by *P2ry12* and *P2ry13*, are the main P2 receptors that regulate microglial surveillance motility and chemotaxis. More specifically, P2Y13 regulates microglial morphology and surveillance (35), whereas P2Y12 mediates directed motility in microglia, process extension and microglia migration toward stimuli including ATP release (34, 41, 88). *P2ry12* and *P2ry13* are strongly downregulated in the transcriptomic studies summarized here (Figure 3), a finding that has been confirmed in mouse and human tissue at the RNA and protein levels (74, 75, 91, 120). Given the functional role of these two receptors, a strong decrease of P2Y13 may contribute to a loss of microglial surveillance function in neurodegeneration that normally supports tissue integrity and downregulation of P2Y12 may affect microglial directed migration, including migration to the plaque site in AD. Furthermore, P2Y12 is important to balance hyperexcitability of neurons, most likely through the involvement of P2Y12 in cell process extension toward the synaptic site (41) and P2Y12 deletion exacerbates experimentally induced epileptic events in mice (121). It is thus tempting to speculate that a reduction of P2Y12 in microglia in AD may contribute to the increased occurrence of epileptic events in AD patients (122).

In addition, brain-derived neurotrophic factor (BDNF) serves, among other functions, as a signal to regulate neuronal activity, since BDNF-mediated disruption of chloride gradient and following disturbance of chloride-dependent GABAergic inhibitory function was reported (123–125). Exocytosis of BDNF is mediated by ATP-evoked P2X4 activation (123, 126–130) and regulated by A2A (118). Decrease of inhibitory signals favors increased neuronal activity and hyperexcitability, eventually causing epileptic seizures (131–134). Both of the mentioned purinergic receptors, P2X4 and A2A, that regulate BDNF release are upregulated in AD [Figure 3, (119)]. The upregulation and following release of BDNF may therefore also contribute to hyperexcitability and epileptic events in neurodegeneration.

Besides, P2X4 is involved in the chemotactic response of microglia, as pharmacological inhibition and downregulation of P2X4 lead to impaired chemotaxis *in vitro* (36). The transcriptomic studies we summarize here provide evidence that *P2rx4* is upregulated in different murine AD models (Figure 3), implicating that it could play a functional role in altering microglial chemotaxis in AD.

In summary, a number of purinergic receptors that regulate microglial morphology, motility and migration are dysregulated in neurodegeneration and may thus constitute pathways that contribute to impairment of these microglial motility functions. It will be important to validate this assumption in functional studies in the future.

Another important microglial cell function that is affected in neurodegeneration is the phagocytic capacity (87), which among many other mechanisms, is regulated by the UDP-sensitive purinergic receptor P2Y6. Interestingly, it has been shown that the ion channel function of P2X4 is inhibited by P2Y6 upregulation. This suggests an interesting mechanism, in which P2RY6 would be key to switch from migratory to phagocytic behavior of microglia (135). Because *P2ry6* is downregulated in several murine AD models (Figure 3), this could mean that UDP-sensitive phagocytosis may be decreased in neurodegeneration. This could also be relevant for a process called “phagoptosis,” i.e., the uptake of live neurons by microglia involving P2Y6 (136). Inhibition of P2Y6 prevents neuronal loss induced by low levels of Aβ, suggesting that microglial phagocytosis could be responsible for neuronal loss (136). However, the absence of P2Y6-dependent inhibition of *ex vivo* phagocytosis in 5xFAD mice indicates that in AD, purinergic receptors other than P2Y6 may regulate microglial phagocytosis (137). Unlike in AD models, *P2RY6* was upregulated in a PD model and in human PD brains, implying an opposite functional impact of P2Y6 in PD (101, 138).

In addition to P2Y6, there is also evidence that P2Y2 contributes to phagocytosis functions in microglia. Specifically, Aβ-induced microglial ATP release enhances phagocytosis of Aβ via P2Y2 activation in cultured cells (103, 139). *In vivo* data confirmed that heterozygous deletion of P2Y2 enhances β-amyloid plaque burden, indicating that downregulation of P2Y2 could contribute to AD pathology (140, 141). Apart from phagocytosis, pinocytosis induced by ATP or UTP is another mechanism that contributes to Aβ uptake by microglia. This process can be accelerated by yet another purinergic receptor, namely P2Y4 (142).

Altogether, since purinergic signaling regulates phagocytosis, a key function of microglia allowing clearance of pathologic protein aggregates, dysregulation of these purinergic pathways is likely to contribute to neurodegenerative disease pathology.

In light of the importance of purinergic signaling for the regulation of many microglia functions, the balance of extracellular purinergic ligands such as ATP, ADP, AMP and adenosine gains in significance. The concentration of extracellular purinergic elements is regulated by purinergic enzymes and transporters. Ectonucleotidases, namely CD39 and CD73, through ATP degradation and adenosine production, modulate microglial migration, microglia morphology and process elongation (114, 143, 144) and reduce phagocytosis (145). It is thus conceivable that downregulation and therefore loss of CD39 function, as observed in the spinal cord of ALS subjects (75) and in transcriptomic data from SOD1 mutant mice and murine amyloidosis models (Figure 3), could have detrimental consequences. Additionally, *Enpp1* in turn is upregulated, while microglial ENT1 and ENT3 are downregulated (Figure 3). This indicates that the regulation of extracellular ligands by ectonucleotidases and by cross-membrane transport may become disturbed in neurodegeneration, although the exact impact on microglia themselves and other CNS cells will require further investigation.

### Astrocytes

Astrocytes become reactive upon neurodegeneration and display morphological, functional, and molecular changes. It has recently been re-confirmed in a consensus statement that reactive astrocytes should not be subclassified into binary categories but defined at multiple levels using morphological, molecular and functional parameters and considering temporal and spatial aspects (6). Interestingly, the presence of reactive astrocytes correlates with the cognitive status in AD (146, 147). This may be due to the fact that astrocytes are involved in a wide variety of functions in the CNS and during neurodegeneration lose some of these properties (Figure 1, right), such as their ability to maintain the integrity of the blood brain barrier, contribute to gliotransmitter release and glutamate uptake. This loss of homeostatic defensive function, which may correlate with astroglial atrophy or “asthenia,” has been attributed to contribute to the propagation of cognitive decline in AD (148). At the same time, astrocytes gain potentially toxic functions and release pro-inflammatory molecules.

Based on the transcriptomic data we assembled here (Figure 2), it appears clear that mouse and human astrocytes show distinct expression patterns. *P2ry2, Enpp2, Enpp5*, and *Gja1* (encoding CX43) are upregulated in AD mouse models, although not consistently across the different studies, while only *Gja1* was found upregulated in human astrocytes in AD (Figure 4). Additionally, *Adora2a, Adora2b, P2y14, Entpd1* (encoding CD39), *Entpd2*, and *Enpp4* are downregulated in AD models or human AD. Furthermore, different sets of purinergic genes were found upregulated (*P2ry6*) or downregulated (*Entpd3, Entpd4, Entpd7*) in an ALS mouse model.

Functionally, the P2Y1 receptor signaling pathway is one of the major purinergic signaling pathways that may contribute to altered astrocyte properties in neurodegeneration, especially in AD. P2Y1 mediates calcium waves in astrocytes, which are linked to gliotransmitter release and synchronization of astrocytic syncytium, which is critical for synaptic plasticity (149–151). Unlike in the transcriptomic studies we summarized here, previous studies showed that astrocytic P2Y1 receptor expression is elevated in AD mouse models and human AD brain, especially in close vicinity to amyloid plaques (151, 152). This spatially restricted expression pattern may explain some of the discrepancies to the transcriptome data. P2Y1 activation has been linked to cognitive decline by enhancing astrocyte hyperactivity (153) and mediating astroglial network defects (151). Acute pharmacological inhibition of P2Y1 allows normalization of such defects (151), highlighting the therapeutical potential of targeting P2Y1 in AD. In particular, P2Y1 inhibitor injected through chronic ICV infusion reduced dystrophic neurite burden, improved astroglial function and long-term potentiation in an AD animal model (152).

Another study that shows discrepancies to the transcriptomic analyses shown here, found increased levels of CD39L1 (encoded by *ENTPD2*) at Braak stage III-IV in AD (154). Since CD39L1 modulates ATP metabolism and is particularly expressed in astrocytes (25), increased CD39L expression would reduce local ATP levels and thus dampen P2Y1-mediated astrocytic hyperactivity in CNS tissue with AD pathology, while its reduced expression, as suggested by the transcriptomic analyses, would have the opposite effect. Further studies are required to resolve these potential effects.

In agreement with the upregulation of *Gja1*/Connexin-43 (CX43) in astrocytes found in the transcriptomic analyses from AD mouse models and human AD brains we summarized here (Figure 4), Gja1 was detected among the astrocytic genes dysregulated in the proximity of amyloid plaques (155). CX43 contributes to ATP release (156). These effects seem to be mediated by amyloid pathology, as amyloid exposure triggers increased expression of CX43, both *in vitro* and in AD models (157–159). Deletion of astroglial CX43 in an AD mouse model was able to reduce astrocyte reactivity, ATP release, plaque-associated neuronal damage and improved synaptic function (160). In both, an *in vitro* and a mouse model of PD, astroglial CX43 was increased following exposure of rotenone, a neurotoxic substance (161, 162). Interestingly, CX43 activity is downstream of P2X7 and contributes to ATP release from astrocytes (163). Using a CX43 inhibitor, reduced α-synuclein deposits and attenuated neuroinflammation in a PD rat model were observed (164), highlighting the therapeutic potential of CX43 in several neurodegenerative diseases. CX43 and PANX1 are downregulated in an ALS mouse model (SOD1 mutant) at pre-symptomatic stage, while being upregulated at symptomatic stage (165), which again suggests that disease-dependent activation stages should be taken into account when designing therapeutic intervention.

Finally, despite weak expression in astrocytes under physiological conditions, adenosine A2A receptor was shown to be increased in astrocytes in both human AD brains (166) and amyloid models (49, 166, 167), although this finding is not supported by the transcriptomic data assembled here (Figure 4). Astrocytic A2A receptor is known for its regulation of glutamate and GABA uptake (49, 50) but also its *in vitro* effect on astrocyte gene regulation (168). Blocking A2A-mediated signaling was sufficient to mitigate memory defects in animal models (166).

Further investigations will be needed in the future to better understand whether A2A is clearly dysregulated in astrocytes or astrocytic subpopulations and whether astrocyte-specific A2A directly impacts astrocyte function in neurodegeneration.

### Oligodendrocytes

In neurodegenerative diseases, oligodendrocytes become progressively damaged from various causes, such as neuroinflammation, direct effects from protein aggregates or oxidative stress, leading to myelin loss, disruption of energy transfer to neurons, and ultimately cell death (169). Regarding oligodendrocytes in neurodegenerative diseases, we could only find few transcriptomic data sets with significant differentially expressed purinergic genes. Of those, *P2rx7* and *Enpp4* genes appear to be upregulated in AD mouse models, and *Adora1* downregulated (61, 68, 72) (Figure 5).

All members of the oligodendrocyte lineage are very sensitive to adenosine and ATP released by neurons or other glial cells as they are able to migrate, differentiate and proliferate upon activation of A1, A2A and P2Y1 receptors through calcium signaling (29, 170–172). In AD, impaired repair of myelin is observed, which was postulated to contribute to disease initiation (173). During neurodegenerative conditions, increased ATP release induces activation of P2X7 in oligodendrocytes, which could serve as an early sensor of neuronal damage, promoting OPC migration *in vivo* (174). Despite several studies pointing out P2X7 implication in different demyelinating context as well as oligodendrocyte death, no experimental data was found in proteinopathies (175–178).

Also, the involvement of oligodendrocyte-specific connexins such as connexin-29 (CX29 or *Gjc3*), connexin-32 (CX32 or *Gjb1*) or connexin-47 (CX47 or *Gjc2*) in a manner similar to CX43 in astrocytes cannot be ruled out (179). A study showed a downregulation of oligodendrocytic CX47 in an AD model, contrasting with the increase of CX43 observed in astrocytes (180). The authors stipulate that such modification could favor astrocyte-astrocyte connection at the expense of astrocyte-oligodendrocyte communication, contributing to oligodendrocyte function impairment. Additionally, CX32 was suggested to be increased in PD and correlated with increased alpha-synuclein uptake (181). Thus, consequences of ATP release from oligodendrocyte connexins and pannexins in neurodegeneration remain to be investigated.

Overall, proliferation and maturation of oligodendrocyte lineage are strongly influenced by purinergic signaling. As such, an imbalanced purinergic signaling upon neurodegeneration could contribute to myelin loss. Noteworthy, most studies on oligodendrocytic purinergic signaling have been conducted on multiple sclerosis and other inflammatory diseases [for review see Welsh and Kucenas (182)]. The lack of data on the present topic should be addressed in the future as P2X7 was found significantly upregulated in oligodendrocytes in AD (61), which could imply a deleterious role in disease progression.

### Purinergic Signaling Crosstalk in Neurodegeneration

Given the importance of purinergic signaling for inter-glial and neuro-glial communication, alterations in cell-specific glial purinergic signaling in neurodegeneration will inevitably lead to disruptions in purinergic inter-cellular crosstalk (Figure 1, right).

For example, although adenosine has been reported to be the main purine metabolite that is elevated in neurodegeneration, at least in AD (56), it cannot be ruled out that opposite changes may take place in a more restricted spatial or temporal manner. For instance, CD39, a critical enzyme for the extracellular hydrolysis of ATP, is downregulated in microglia in AD (Figure 3) (41), especially in the vicinity of amyloid-beta plaques. This could have two immediate consequences: firstly, a local lack of adenosine production, which would reduce adenosine receptor activation, and secondly, high local levels of ATP, which could trigger microglial P2X7 receptor. In line with this, global CD39 deletion was shown to increase ATP and decrease adenosine in the CSF (183). Furthermore, global and microglia-specific *Entpd1*/CD39 deletion is associated with neuronal hyperactivity and increased vulnerability to epileptic events, mediated by a decreased activation of the inhibitory neuronal adenosine A1 receptor (41, 183). The reduced expression of astrocytic *ENTPD1*/CD39 in human AD cases (Figure 4), could further amplify these events. Additionally, a local ATP increase would trigger other P2 receptors such as astrocytic P2Y1, which would further impair neuronal synchronicity through astrocytic hyperactivation (151, 152). Adding further complexity, the ectonucleotidase ENPP1 is increased in microglia in AD (Figure 3). Whether this can–at least partially–compensate for decreased CD39-mediated ATPase activity and whether this affects astrocytic and oligodendrocyte functions has not been investigated. Hopefully, future studies will be able to answer these questions.

Furthermore, there is evidence that neuro-glial communication is affected by upregulation of glial purinergic receptors in neurodegeneration. For instance, microglial upregulated P2X7 (184–186) leads to an exacerbation of a pro-inflammatory microglial phenotype and increased release of proinflammatory molecules, which disrupt synaptic communication (187–189). Pro-inflammatory molecules like chemokines or reactive oxygen species also affect oligodendrocytes and astrocytes, causing oligodendrocyte cell death (190–192) and astrocytic reactivity (6), leading to further cell and network impairment. P2X7 is also upregulated in oligodendrocytes and has been implicated in oligodendrocytic cell death, which would critically alter inter-neuron communication and neuronal metabolic support. Furthermore, increased astrocytic P2Y1 in neurodegeneration, which is associated with astrocyte hyperexcitability, network synchronicity loss and increased ATP/glutamate release (152, 193) could in turn reinforce P2X7 activation in microglia and oligodendrocytes. Some studies have also reported increased A2A receptor expression in astrocytes, which has been shown to reduce glutamate intake and increase GABA intake (49, 50), thus favoring an excitotoxicity state, which is detrimental for synaptic function and reinforces reactive phenotypes of microglia and astrocytes.

In summary, increased ligand availability (ATP and adenosine) together with a distinct set of activated glial purinergic signaling pathways (P2X7, P2Y1, A2A) and loss of homeostatic neuronal and glial purinergic signaling pathways (A1, P2Y12, P2Y13) during neurodegeneration may alter the balance of purinergic signaling homeostasis. This in turn could lead to more cellular damage (excitotoxicity, neuroinflammation, myelin loss), loss of homeostatic functions (reduced energy delivery, reduced trophic factors, impaired synchronicity between networks) and promote protein aggregation (reduced phagocytosis of protein aggregates), thus ultimately favoring the progression of disease pathology in neurodegenerative diseases.

## Therapeutical Perspectives

Recent advances have highlighted the beneficial effect of targeting purinergic signaling in neurodegenerative diseases, notably with the authorization of istradefylline, a selective antagonist of A2A receptors, in the co-treatment of Parkinson's disease in the USA in 2019 (194), following the approval in Japan in 2013. These first steps show that targeting purinergic signaling can be safe for use in neurodegenerative diseases and help to slow disease progression. Additionally, several drugs targeting purinergic signaling have gone into clinical trial in order to treat inflammatory diseases like rheumatoid arthritis, that are beyond the scope of this article [for review see Antonioli et al. (195)]. Noteworthy, proteinopathies are often associated with chronic low-grade neuroinflammation, reinforcing the potential benefits of using drugs targeting purinergic signaling (196).

P2X7 is one of the most evident targets of glial purinergic signaling in neurodegeneration, being upregulated in both microglia and oligodendrocytes at the protein levels (61). It was postulated that P2X7 is also expressed in neurons, but this was disproved by unbiased studies and the use of specific tools (197). Indeed, activation of P2X7 requires high level of ATP, which is found in neurodegeneration (198). It has been hypothesized that P2X7 acts as an early sensor, which represents a prerequisite for glial response to insults. However, chronic activation of P2X7 results in cell death, contributing to disease progression. Blocking P2X7 mitigates amyloid burden in AD models and improves synaptic plasticity, integrity and memory (94, 108, 199). Additionally, increased motor neuron survival and decreased microgliosis and inflammatory markers were shown after P2X7 inhibition at late pre-onset in SOD1 mutant mice (110). Furthermore, using P2X7- or P2Y6-selective antagonists, BBG or MRS2578 respectively, in an animal model of PD, neuroprotection and a reduced microglia reactive phenotype were observed (101, 200).

Adenosine receptors also represent suitable targets for glial modulation. Administration of the A2A receptor antagonist preladenant partly decreased *ex vivo* hyperactive motility around Aβ plaques in the 5xFAD amyloidosis model (89). In addition, preladenant restored microglial process extension toward tissue damage in the MPTP-induced PD model (201). Several studies have shown increased astrocytic A2A in pathological context such as AD, which suggests an abnormal function (166, 167). Additionally, A2A has also been studied as a therapeutical target in neuroinflammatory conditions involving myelin loss and T cell activation (202). Altogether, these results suggest that adenosine receptors like A2A could be targeted therapeutically to improve the disease-associated phenotype of glial cells in proteinopathies.

Apart from purinergic receptors, several studies have highlighted elevated astrocytic CX43 in neurodegenerative diseases, leading to increased ATP release. Together with other nucleotide transporter such as pannexin-1, they could be targets of choice in order to decrease ATP release and aberrant purinergic signaling in neurodegeneration (203).

## Conclusion

As outlined here, purinergic signaling in neurodegeneration is not only altered in neurons, but in all CNS cell types, including glial cells. This highlights the potential to target purinergic signaling in a multi-cellular fashion. However, to develop this as a valuable strategy in the future, many functional aspects of purinergic signaling in glial cells need to be further elucidated. In particular, purinergic signaling in astrocytes and oligodendrocytes have remained ill-defined, as well as purinergic signaling pathways in proteinopathies involving FUS or TDP-43 aggregates. Furthermore, novel tools are needed that help to better define the cell-specific and the spatio-temporal aspect of purinergic signaling in neurodegeneration. These challenges will need to be faced in the future to better understand this fascinating system, as within it potentially lies the hopes of tomorrow's treatments.

## Author Contributions

MP, AG, SK, AH, and KC wrote the manuscript. MP, AG, SK, DB, AH, and KC reviewed the manuscript. All authors contributed to the article and approved the submitted version.

## Conflict of Interest

The authors declare that the research was conducted in the absence of any commercial or financial relationships that could be construed as a potential conflict of interest.
